# Geroscience‐guided repurposing of FDA‐approved drugs to target aging: A proposed process and prioritization

**DOI:** 10.1111/acel.13596

**Published:** 2022-03-27

**Authors:** Ameya S. Kulkarni, Sandra Aleksic, David M. Berger, Felipe Sierra, George A. Kuchel, Nir Barzilai

**Affiliations:** ^1^ 2006 Institute for Aging Research Albert Einstein College of Medicine Bronx New York USA; ^2^ 2006 Department of Medicine (Endocrinology and Geriatrics) Albert Einstein College of Medicine Bronx New York USA; ^3^ Department of Medicine (Hospital Medicine) Montefiore Medical Center and Albert Einstein College of Medicine Bronx New York USA; ^4^ 36760 Centre Hospitalier Universitaire de Toulouse Toulouse France; ^5^ 12227 UConn Center on Aging University of Connecticut School of Medicine Farmington Connecticut USA; ^6^ Present address: AbbVie Inc. North Chicago IL 60064 USA.

**Keywords:** aging, clinical trials, drug repurposing, geroscience, preclinical studies

## Abstract

Common chronic diseases represent the greatest driver of rising healthcare costs, as well as declining function, independence, and quality of life. Geroscience‐guided approaches seek to delay the onset and progression of multiple chronic conditions by targeting fundamental biological pathways of aging. This approach is more likely to improve overall health and function in old age than treating individual diseases, by addressing aging the largest and mostly ignored risk factor for the leading causes of morbidity in older adults. Nevertheless, challenges in repurposing existing and moving newly discovered interventions from the bench to clinical care have impeded the progress of this potentially transformational paradigm shift. In this article, we propose the creation of a standardized process for evaluating FDA‐approved medications for their geroscience potential. Criteria for systematically evaluating the existing literature that spans from animal models to human studies will permit the prioritization of efforts and financial investments for translating geroscience and allow immediate progress on the design of the next Targeting Aging with MEtformin (TAME)‐like study involving such candidate gerotherapeutics.

## Graphical abstract

Geroscience‐guided approaches seek to delay the onset and progression of chronic conditions by targeting the pathways of aging. This approach is likely to improve overall health in old age by addressing aging—the largest risk factor for the leading causes of morbidity. Here, we propose the creation of a standardized process for evaluating FDA‐approved drugs for their geroscience potential, permitting the prioritization of efforts and investments for translating geroscience discoveries.

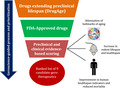

**Abbreviations**
ACEi: angiotensin‐converting enzyme inhibitor
ACS: Acute Coronary Syndrome
AD: Alzheimer’s disease
ADAS‐cog: Alzheimer's Disease Assessment Scale–Cognitive Subscale
ARB: angiotensin receptor blocker
BCC: basal cell carcinoma
BEZ235: dactolisib
BMD: bone mineral density
BP: blood pressure
CAD: Coronary Artery Disease
cIMT: coronary (artery) intimal media thickness
CKD: chronic kidney disease
CRC: colorectal cancer
CV: cardiovascular
CVA: cerebrovascular accident
DM: diabetes mellitus
ESRD: end‐stage renal disease
GFR: glomerular filtration rate
HF: heart failure
HR: hazard ratio
HRCT: high‐resolution chest CT
HTN: hypertension
IFN: interferon
ILD: interstitial lung disease
IPF: idiopathic pulmonary fibrosis
IRR: incidence rate ratio
MACE: major adverse cardiovascular event
MCI: mild cognitive impairment
MI: myocardial infarction
MMSE: mini mental status evaluation
OR: odds ratio
RAD001: everolimus
RCT: randomized controlled trial
RR: relative risk
SASP: senescence‐associated secretory phenotype
SRR: standardized rate ratio
SU: sulfonylurea
T2D: diabetes mellitus, type 2

## 1 INTRODUCTION

Geroscience represents a novel paradigm whereby biological aging is recognized as the major modifiable driver of age‐related diseases and other late‐life conditions (Burch et al., 2014; Kennedy et al., 2014; López‐Otín et al., 2013). Widespread clinical use of geroscience‐guided interventions could transform the public health landscape because the ability to target biological aging as a risk factor could simultaneously delay the onset and progression of multiple conditions, thereby enhancing health, function, and independence in late‐life. A corollary is that targeting this biology will affect human healthspan (the portion of lifespan free of major disease and disability) most profoundly, and with a better prognosis than the current model of addressing one disease at a time. Geroscientists agree on several criteria defining distinct yet interrelated molecular and cellular hallmarks of aging that include (1) the measurable systemic or tissue‐specific biological process should be altered during aging; (2) its disruption should have negative consequences in both lifespan and healthspan; and (3) when positively modulated, it should extend both lifespan and healthspan in preclinical models (Huffman et al., 2016; Kritchevsky & Justice, 2020). Currently approved and accepted tools to target aging in humans are restricted to diet and exercise, which seem to influence multiple hallmarks of aging. On the contrary, advances in geroscience are spurring the development of gerotherapeutics (pharmacological interventions that promote health by directly or indirectly targeting the aging hallmarks, in addition to the disease itself) which show efficacy toward multiple age‐related conditions. Interventions that influence these hallmarks at the cellular level toward more youthful function appear to lead to similarly favorable functional changes at the organ and systemic level.

While aging is unequivocally the major risk factor for age‐related diseases, regulatory bodies around the world, such as the FDA or EMA, do not yet recognize geroscience‐guided clinical outcomes as a path to regulatory approval. This is in part because the processes for validating specific compounds or combinations of compounds for their ability to delay the onset and progression of multiple chronic diseases have not yet been delineated in humans. Without regulatory approval, insurers will not pay for such treatments, which disincentivizes pharmaceutical companies from developing geroscience‐guided approaches, simply because there is no path for them to develop a viable business plan. Therefore, there is an urgent need to demonstrate, in a well‐designed clinical trial, that a cluster of age‐related diseases can be significantly delayed by repurposing existing or developing novel gerotherapeutics.

Targeting Aging with MEtformin (TAME) is such a study that has been under development for the last few years, and whose basic principles have been developed in consultation with the FDA (Barzilai et al., 2016). Metformin was initially used in the 1950s to prevent influenza and malaria, but it was also noted to lower glucose levels in people with diabetes without triggering hypoglycemia, leading to it becoming the treatment of choice for type 2 diabetes (T2D). In addition to its well‐documented benefits on diabetes, clinical and observational studies have linked metformin to beneficial effects on multiple age‐related diseases, including cardiovascular disease (CVD), cancer, Alzheimer's disease, and mild cognitive impairment, as well as reduced mortality (Barzilai et al., 2016; Campbell et al., 2018; Novelle et al., 2016). TAME was designed by a group of geroscientists as a robust proof‐of‐principle study, whereby the primary outcome of the placebo‐controlled, double‐blinded trial will be the “time to event” of a cluster of age‐related diseases consisting of cardiovascular events, cancer, cognitive decline, dementia, and death. In this manner, TAME is planned to provide a template for the design of future studies evaluating other FDA‐approved drugs that are repurposed as gerotherapeutics (Espeland et al., 2017; Justice et al., 2018).

We believe that efforts to test and repurpose existing, safe gerotherapeutics should be extended beyond TAME, not only to increase the number of drugs potentially available to target aging in humans but also to mitigate the risks to the field, should any such trials fail to reach their desired outcome. The purpose of this paper is to gather available evidence in the literature that supports and ranks potential gerotherapeutics and to help clinical investigators, geroscientists, not‐for‐profit foundations, and governments to accelerate research and clinical trials to test their efficacy and safety. Importantly, our mission is for such drug candidates to become clinically available relatively soon, and thus, we limit our analyses only to potential gerotherapeutics that are already FDA‐approved for other clinical indications.

We sought to identify such FDA‐approved drugs or classes of drugs that had at least one publication showing extension of lifespan in rodents and data in humans suggesting the highest chance of success if tested in a well‐controlled TAME‐like clinical trial. We developed a 12‐point prioritization scale that assigns equal points for the preclinical and clinical evidence for each of these candidates. Points on the preclinical side were assigned for effects on the hallmarks of aging, improvement in healthspan and extension of lifespan in rodents as part of the NIA’s Interventions Testing Program (ITP), a well‐characterized, multicentered study to evaluate gerotherapeutics, as well as non‐ITP rodent lifespan studies (Harrison et al., 2014; Nadon et al., 2017). On the clinical side, points were assigned for observed healthspan outcomes extending beyond the diseases targeted by the drug, and mortality from any cause or off‐target diseases, with a scale where interventional studies weigh more than observational studies. Here, we outline the nature of the process and priority scoring designed to assess the likelihood that a specific gerotherapeutic may be successful in a clinical study.

## 2 METHODS

### 2.1 Identification of potential gerotherapeutics among FDA‐approved drugs

**FIGURE 1:**
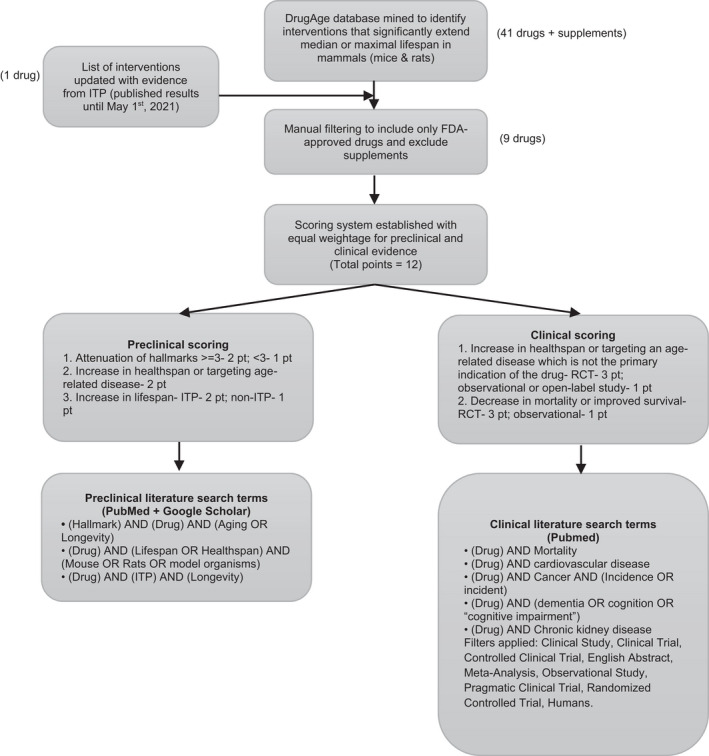
Workflow to select candidate gerotherapeutics and evaluate preclinical and clinical evidence for future geroscience‐guided clinical trials. Number of interventions after each step of selection are indicated in parentheses

We conducted mining of the DrugAge (Build 3) database for drugs that extend lifespan (Barardo et al., 2017; detailed in Figure 1). Results were then filtered for rodents (mice and rats) in studies reporting statistically significant increased median and/or maximum lifespan as the inclusion criteria (*n* = 41 drugs or supplements). This approach meant that drugs that have shown some clinical benefits but have not shown extension of lifespan in preclinical rodent models were excluded from further analysis. These findings were then updated with drugs tested in the ITP since 2019 (DrugAge Build 3 was released in July 2019). Nutraceutical/supplemental products were excluded, and only FDA‐approved drugs were included (*n* = 9 drugs). Search was then split into preclinical (more detail on lifespan, measures of healthspan and hallmarks of aging) and clinical (healthspan and mortality) using the search terms and datasets as described in Figure 1. The search terms for clinical outcomes were based on the non‐communicable diseases among the 10 leading causes of death in persons age 65 and older in the US (CDC, 2017).

### 2.2 Ranking gerotherapeutics

An ordinal 12‐point scale was equally divided between basic and clinical studies (6 points each). Thereby, promising gerotherapeutics that have significant basic geroscience rationale, yet currently lack supportive data from human subject studies were not penalized.

### 2.3 Preclinical scoring

Of the 6 points evaluating basic or preclinical factors, up to 2 points were assigned each for targeting hallmarks of aging, improving preclinical healthspan and preclinical lifespan each, with the breakdown as follows: (i) 1 point assigned for less than 3 hallmarks, 2 points assigned for 3 or more hallmarks; (ii) 2 points assigned for effect on healthspan parameters; (iii) 1 point assigned for lifespan tested outside ITP, 2 points assigned for a significant increase in lifespan within ITP (López‐Otín et al., 2013).

### 2.4 Clinical scoring

Out of the 6 points assessing clinical considerations, up to 3 points were assigned to healthspan and 3 points for mortality data, as follows: (i) Healthspan: drug needed to demonstrate that it targeted at least one age‐related disease/pathologic process which it was not intended to treat, with 1 point assigned for observational studies and 3 points for interventional, randomized controlled trials (RCTs); (ii) mortality: drug needed to demonstrate that it reduced all‐cause mortality or death from a disease which it was not intended to treat, with 1 point assigned for observational studies and 3 points assigned for RCTs.

## 3 RESULTS

Using the process detailed in Figure 1, we were able to prioritize 9 drug classes (Table 1). SGLT2 inhibitors (SGLT2i), a relatively new drug class, was the only one to receive the maximum score, owing to not only its robust effects on improving rodent healthspan and lifespan (including ITP) but also strong evidence for the extension of healthspan and reduction of mortality in humans. Metformin was next on the list, and it received a submaximal score, due to negative findings for rodent lifespan extension in ITP. Acarbose, rapamycin/rapalogs, and methylene blue (MB) all had strong preclinical data and promising findings for human healthspan (the latter being the most robust for acarbose), but sparse clinical data for human mortality. Angiotensin‐converting enzyme inhibitors (ACEi) and angiotensin receptor blockers (ARBs) were found to extend preclinical healthspan and lifespan (outside of ITP) and had robust effects on extending human healthspan, but the studies on human mortality, while abundant, were predominantly negative. The last three drugs on our list, senolytics‐ Dasatinib + Quercetin (D + Q), aspirin, and N‐acetyl cysteine (NAC), all had strong preclinical data, but their effects on human healthspan and mortality have not yet been assessed in clinical studies or appropriate doses/populations. Obviously, future studies may change the priority order for drugs that did not receive points due to the paucity of clinical data.

**TABLE 1:** Ranking of FDA‐approved drugs as potential gerotherapeutics based on scoring (out of 12) for preclinical and clinical evidence

Gerotherapeutics	Hallmarks of aging	Preclinical healthspan	Preclinical lifespan	Human healthspan	Human mortality	Score (out of 12)
SGLT‐2 inhibitors	2	2	2	3	3	12
Metformin	2	2	1	3	3	11
Acarbose	2	2	2	3	0 (Not assessed)	9
Rapamycin/rapalogs	2	2	2	3*	0 (Not assessed)	9
Methylene blue	2	2	2	3*	0 (Not assessed)	9
ACEi/ARB	2	2	1	3	0	8
Dasatinib + (quercetin)	2	2	1	1	0 (Not assessed)	6
Aspirin	2	2	2	0 (Not assessed)	0 (Not assessed)	6
N‐acetyl cysteine	1	2	2	0 (Not assessed)	0 (Not assessed)	5

### 3.1 Preclinical results

#### 3.1.1 Rodent lifespan

Gerotherapeutics in preclinical models were primarily evaluated for their ability to extend rodent lifespan (Table 2). Robust evidence from ITP suggests that SGLT2i (canagliflozin), acarbose, rapamycin, MB, and aspirin extend either median and maximum lifespan or median lifespan alone in genetically heterogeneous UM‐HET3 mice, generally in a sexually dimorphic manner (Harrison et al., 2014; Nadon et al., 2017). SGLT2i canagliflozin extended median survival by 14% and the age for 90th percentile survival by 9% in male mice (Miller et al., 2020). Acarbose extended median lifespan by 22% in males and only 5% in females, while rapamycin exhibits a dose‐dependent response by increasing median lifespan by 16%–26% in females and 13%–23% in males (Harrison et al., 2014, 2019; Miller et al., 2011, 2014; Strong et al., 2016, 2020). Although less rigorous than ITP, individual non‐ITP studies have also identified metformin, ACEi/ARB, senolytics D + Q to have a lifespan‐extending effect in mice (Martin‐Montalvo et al., 2013; Santos et al., 2009; Xu et al., 2018).

**TABLE 2:** Preclinical evidence for candidate gerotherapeutics in improving lifespan and healthspan and attenuating hallmarks of aging

Gerotherapeutics	Effects on model organism lifespan	Effects on healthspan and age‐related diseases in preclinical models	Hallmarks of Aging			
			Macromolecular Damage / Adaptation to Stress	Epigenetic effects / Stem Cell renewal and regeneration	Proteostasis / Inflammation / Senescence	Metabolism
SGLT2 inhibitors	ITP: Canagliflozin ↑ median lifespan by 14% in males (Miller et al., 2020)	Dapagliflozin ↓ atherosclerosis with macrophage infiltration in diabetic ApoE −/− mice (Leng et al., 2016)	Dapagliflozin restores Calcium uptake and prevents age‐associated Calcium build up in the mitochondria of cardiomyocytes (Olgar et al., 2020)	No applicable studies	Empagliflozin reactivates glomerular autophagy in db/db mice (Korbut et al., 2020)	Dapagliflozin ↑ cardiac function and glucose tolerance in IR rats with metabolic syndrome via ↑ mitochondrial function and oxidative stress (Durak et al., 2018) Empagliflozin ↑ AMP/ATP ratio, AMPK and ↓ mitochondrial fission (Zhou et al., 2018) Empagliflozin ↓ mTORC1 in diabetic mouse kidneys (Tomita et al., 2020)
Metformin (pre‐2020 studies summarized previously (Kulkarni et al., 2020))	5.83% ↑ in mean lifespan of 84‐weeks‐old males (Martin‐Montalvo et al., 2013)	↑ cognitive function, ↓ microglial activation in 18‐mo‐old male mice (Kodali et al., 2021) ↑ motor symptoms in mouse model of Parkinson's disease via regulation of astrocytes transcriptome (Ryu et al., 2020) ↓ cartilage degeneration and chondrocyte aging in mouse model of osteoarthritis (X. Feng et al., 2020)	↑ mitochondrial function and ↓ endoplasmic reticular stress in aged mouse hearts (Q. Chen et al., 2021) ↓ ROS and RNS via ↑ FOXO3 in human immune cells (Hartwig et al., 2021) ↓ CKD‐induced DNA damage (Kim et al., 2021)	↑ recruitment of neural stem cells, neurogenic potential, brain vascularization and cerebral angiogenesis in aged mouse brain (X. Zhu et al., 2020) ↓ senescence in mesenchymal stem cells (Kim et al., 2021) ↓ senescence in dental pulp stem cells via ↓ miR‐34a‐3p and ↑ CAB39 (S. Zhang et al., 2021)	↑ autophagy in hippocampus and ↓ pro‐inflammatory cytokines in 18‐mo‐old male mice (Kodali et al., 2021) ↓ leaky gut and inflammation via ↑ goblet cell mass and mucin production and modulating the gut microbiome (Ahmadi et al., 2020) ↓ hydrogen peroxide induced senescence in retinal pigment endothelium cells with ↑ autophagy; and human lens epithelial B3 cells (C. Zhang et al., 2020; Zhao et al., 2020)	Regulation of UPR via AMPK/ERK1/2 pathway to attenuate age‐related hearing loss, cell apoptosis and neurodegeneration in old rats (Cai et al., 2020)
Acarbose	ITP: 22% ↑ in median lifespan (males) and 5% ↑ in (females) (Harrison et al., 2014, 2019; Strong et al., 2016)	↓ Incidence of lung tumors (males), ↓ Liver degeneration (both sexes) and ↓ Glomerulosclerosis (females) (Harrison et al., 2019) ↓ Age‐related behavioral and biochemical changes in SAMP8 mice (Tong et al., 2015) ↓ Age‐related memory impairment (Yan et al., 2015) ↓ tumor burden and hematocrit in Apc +/Min mouse model of intestinal tumorigenesis at the higher dose (Dodds et al., 2020)	No applicable studies	↓ PDX−1 methylation in beta‐cells reverting T2DM in db/db mice (D. Zhou et al., 2021)	Modulation of the gut microbiome with ↑ fecal short‐chain fatty acids (Smith et al., 2019) ↓ ER stress response associated with benzene‐induced inflammation in glial cells (Debarba et al., 2020)	↓ Insulin (males only) and ↓ IGF1 (males and females) (Harrison et al., 2014) ↓ postprandial glucose (Harrison et al., 2019) ↓ Age‐associated cardiac lipids including lysophospholipids (Herrera et al., 2020)
Rapamycin and Rapalogs	ITP: Median lifespan‐ 13–23% ↑ (dose‐dependent) in male mice; 16–26% ↑ (dose‐dependent) in female mice; Max lifespan‐ 8% ↑ in male mice; 5–11% ↑ in female mice (Miller et al., 2014)	↓ cognitive decline, ↓ retinopathy, ↓ myocardial alterations, ↓ liver degeneration, ↓ endometrial hyperplasia and ↑ physical activity (Lamming et al., 2012) ↓ Reactive Oxygen Species in human corneal endothelial cells (Shin et al., 2011) ↓ loss of cognition in mouse models of Alzheimer's Disease (Kaeberlein & Galvan, 2019; Spilman et al., 2010) ↓ frailty, ↑ long‐term neuromuscular coordination, memory, and tissue architecture (Correia‐Melo et al., 2019)	↓ oxidative stress induced damage in erythrocytes (Singh et al., 2016) ↓ DNA‐damage accumulation and improved nuclear morphology in fibroblasts from Werner's syndrome (Saha et al., 2014)	Delay age‐related decline in HSCs and restoration of hematopoeisis (C. Chen et al., 2010) ↑ intestinal stem cell renewal, Paneth cell niche via mTORC1 inhibition (Yilmaz et al., 2012) Retention of stemness and youthful phenotype in adult stem cells (Lamming et al., 2012)	Improvement in gut health and muscle proteostasis in drosophila mediated by ↑ autophagy (Schinaman et al., 2019) ↑ autophagy in human neuroblastoma cell lines (Lin et al., 2018) ↓ SASP in a Nrf‐independent manner in senescent cells (Correia‐Melo et al., 2019; R. Wang et al., 2017) ↓ IL1A and NFKB signaling thereby ↓ SASP via mTORC1‐dependent mechanism (Laberge et al., 2015) ↓ adipose tissue inflammation (Paschoal et al., 2017)	↓ TOR signaling in yeast, worms, flies, mice and humans (Blagosklonny, 2019) Reversal of IR via dephosphorylation of IRS1 (Leontieva et al., 2014)
Methylene Blue	ITP: Median lifespan‐ 0.7% ↑ and max lifespan‐ 6% ↑ in females (Harrison et al., 2014)	Improvement in brain ABAD function and ↓ A‐beta in neuroinflammatory mouse model (Zakaria et al., 2016) ↓ Tau accumulation in P301L Tau transgenic mice (Hosokawa et al., 2012) ↓ nuclear and mitochondrial abnormalities in progeroid mouse model (Xiong et al., 2016)	Improved mitochondrial respiration and ↓ reactive oxygen species and oxidative stress in diabetic rat hearts (Duicu et al., 2017; Xiong et al., 2016)	No applicable studies	Delays cellular senescence (Atamna et al., 2008; Xiong et al., 2017)	No applicable studies
ACEi and ARBs	↑ rat lifespan with long‐term enalapril treatment (Santos et al., 2009)	Enalapril attenuates frailty in middle‐aged and older female mice and older male mice (Keller et al., 2019) ↓ angiotensin‐induced atherosclerosis and vascular inflammation in mice (da Cunha et al., 2005)	No applicable studies	No applicable studies	↑ antioxidant defenses in mouse tissues treated with enalapril and captopril (de Cavanagh et al., 1997) ↑ autophagy in prostate cancer cells (Woo & Jung, 2017)	No applicable studies
Dasatinib + Quercetin (Senolytics)	Biweekly treatment in 24‐month‐old mice ↑ median lifespan by 36% (Xu et al., 2018)	↓ A‐beta induced oligodendrocyte senescence, ↓ plaque load and inflammatory cytokines and ↑ cognitive function in young mice (P. Zhang et al., 2019) ↓ senescent cell burden in liver, irradiated muscle; ↑ cardiac function and healthspan indicators in movement dysfunctions in old mice (Xu et al., 2018; Y. Zhu et al., 2015) ↓ senescent cells in aged and hypercholesteremic mice and ↑ vasomotor functions in mice (Roos et al., 2016) ↓ senescent cells in idiopathic pulmonary fibrosis (Schafer et al., 2017) ↓ age‐related bone loss (Farr et al., 2017) ↓ uterine age‐related dysfunction and fibrosis (Cavalcante et al., 2020)	↓ in cf‐mtDNA and associated inflammation with ↑ survival of old cardiac allografts (Iske et al., 2020) ↓ radiation ulcers caused due to radiotherapy and radiation induced DNA damage (H. Wang et al., 2020)	No applicable studies	↓ intestinal senescence and inflammation with a modulation of gut microbiome (Saccon et al., 2021) ↓ pro‐inflammatory cytokine release in human adipose tissue (Xu et al., 2018)	↑ insulin sensitivity and glucose tolerance in obese mice (Sierra‐Ramirez et al., 2020)
Aspirin (dose‐specific effect)	ITP: (21 mg/kg)‐ Median lifespan 8% ↑ in males (Strong et al., 2008)	↓ amyloid plaque pathology via lysosomal biogenesis in Alzheimer's mouse model (Chandra et al., 2018) ↓ colorectal tumor incidence in a microbiome‐dependent manner (Zhao et al., 2020) Prevention of age‐related endothelial dysfunction (Bulckaen et al., 2008)	Protection against UVB‐induced DNA damage in melanocytes and keratinocytes in C57B6 mice (Rahman et al., 2021) ↓ in markers of oxidative stress (Bulckaen et al., 2008) ↓ Reactive Oxygen Species and decreased onset of senescence in endothelial cells (Bode‐Böger et al., 2005)	Suppression of age‐related and CC‐hypermethylation in colon (Noreen et al., 2020)	↑ autophagy in C. elegans via EP300 inhibition (Pietrocola et al., 2018) ↓ senescence in doxorubicin‐induced models of senescent human and mouse fibroblasts (M. Feng et al., 2019) ↓ necrosis in astrocytes with ↓ pro‐inflammatory mediators IL‐beta, TNF‐alpha and NFKB signaling and ↑ anti‐inflammatory responses with PPAR‐gamma signaling (Jorda et al., 2020)	↑ AMPK, ↓ mTOR signaling and recapitulating metabolic effects of caloric restriction (Pietrocola et al., 2018)
N‐acetyl Cysteine	ITP effect only in UM mice(could be due to inadvertent dietary restriction) (Flurkey et al., 2010)	↓ age‐related hearing loss, memory decline, spatial memory deficits and oocyte aging in mice (Costa et al., 2016; Liu et al., 2012; Marie et al., 2018; More et al., 2018) (Marie et al., 2018; Costa et al., 2016; Lamming et al., 2012; More et al., 2018)	↓ oxidative stress and neurodegeneration in rat brain (Garg et al., 2018)	No applicable studies	No applicable studies	No applicable studies

#### 3.1.2 Rodent healthspan

In addition to lifespan effects, we evaluated the evidence of potential gerotherapeutics on improving healthspan in preclinical models of aging and age‐related diseases, where the action of the drug was attributed to their non‐primary target or mechanism. Metformin displayed strong evidence in targeting conditions that were beyond its antihyperglycemic function (Barzilai et al., 2016; Kulkarni et al., 2018, 2020). In osteoarthritic mice, it moderated cartilage degeneration and chondrocyte aging (Feng et al., 2020). Similarly, SGLT2i and acarbose demonstrated effects beyond their glucose‐lowering potential by lowering the incidence of atherosclerosis in diabetic ApoE −/− mice with the former, and that of lung tumors, liver degeneration, and glomerulosclerosis with the latter (Han et al., 2017; Harrison et al., 2019; Liu et al., 2021). In mouse models of neurodegeneration, metformin, acarbose, rapamycin, MB, aspirin, and NAC improved cognitive and motor functions and attenuated age‐related memory impairments and behavioral changes (Cao et al., 2012; Chandra et al., 2018; Hosokawa et al., 2012; Kaeberlein & Galvan, 2019; Kodali et al., 2021; Lamming et al., 2012; Martínez et al., 2000; Ryu et al., 2020; Spilman et al., 2010; Tong et al., 2015; Yan et al., 2015; Zakaria et al., 2016).

Complementary to its effects on lifespan, rapamycin improved mouse healthspan by reducing the incidence of retinopathy, myocardial alterations, liver degeneration, and endometrial hyperplasia while also scavenging reactive oxygen species (ROS) in corneal endothelial cells (Lamming et al., 2012; Shin et al., 2011).

Rapamycin, metformin, and ACEi enalapril are also shown to impact indicators of frailty, including motor and physical performance, grip strength, stride length, resistance to muscle fatigue as well as decreased incidence of kyphosis index and cataracts in middle‐aged and older mice (Correia‐Melo et al., 2019; Keller et al., 2019; Palliyaguru et al., 2019).

The effects of senolytics D + Q are primarily attributed to lowering the burden of senescent cells or downregulating inflammation in older mice and thereby preventing aging‐related cognitive, mobility declines, frailty, vasomotor, uterine dysfunction, or fibrosis (Cavalcante et al., 2020; Farr et al., 2017; Roos et al., 2016; Schafer et al., 2017; P. Zhang et al., 2019).

#### 3.1.3 Attenuation of hallmarks of aging

To assess the molecular utility of gerotherapeutics in targeting fundamental mechanisms of aging, we subsequently evaluated their actions on hallmarks of aging. By grouping similar pathways together, the seven pillars of aging were classified into four (1) Macromolecular damage and adaptation to stress; (2) epigenetic effects, stem cell renewal and regeneration; (3) proteostasis, inflammation (and senescence); and (4) metabolism (Kennedy et al., 2014; López‐Otín et al., 2013). Importantly, drugs well‐studied from the aging perspective such as metformin, rapamycin, and aspirin have substantial evidence of targeting hallmarks of aging, as compared to relatively less studied or newly approved drugs including SGLT2i, senolytics, and NAC.

We have previously elaborated on metformin's impact on attenuating hallmarks of aging (Kulkarni et al., 2020). Further investigation on other gerotherapeutics indicates that they target common mechanisms within each hallmark of aging, which includes scavenging of ROS and thereby oxidative stress by metformin in human immune cells, rapamycin in erythrocytes, MB in rat hearts, aspirin in endothelial cells, and NAC in the brain (Duicu et al., 2017; Garg et al., 2018; Hartwig et al., 2021; Jorda et al., 2020; Singh et al., 2016). Moreover, metformin, rapamycin, and aspirin further protect against DNA damage either via lowering DNA‐damage accumulation (rapamycin and aspirin) or upregulating DNA‐damage response (metformin) (Chen et al., 2011; Kulkarni et al., 2020; Rahman et al., 2021; Saha et al., 2014). SGLT2i improves mitochondrial function by preventing calcium buildup, while metformin and MB regulate the electron transport chain (Chen et al., 2021; Duicu et al., 2017; Olgar et al., 2020; Xiong et al., 2017).

Many gerotherapeutics exhibit anti‐inflammatory, senolytic/senomorphic, and proteostasis‐regulating properties. Metformin, rapamycin/rapalogs, ARBs, and aspirin increased autophagy in a tissue‐specific manner, SGLT2i empagliflozin reactivates glomerular autophagy in db/db mice (Bharath et al., 2020; Castoldi et al., 2020; Kodali et al., 2021; Korbut et al., 2020; Lin et al., 2018; Pietrocola et al., 2018; Schinaman et al., 2019; Woo & Jung, 2017; Zhao et al., 2020). Metformin, rapamycin, senolytics, and aspirin are seen to lower peripheral and tissue‐specific pro‐inflammatory cytokines (Jorda et al., 2020; Kodali et al., 2021; Kulkarni et al., 2020; Laberge et al., 2015; Paschoal et al., 2017; Saccon et al., 2021; Zhang et al., 2019). Recent evidence also suggests the role of metformin and acarbose in modulating the gut microbiome, thereby suppressing inflammation (Ahmadi et al., 2020; Smith et al., 2019). In addition to senolytics, metformin, rapamycin, MB, and aspirin have also been shown to lower senescence‐associated secretory phenotype (SASP) levels (Atamna et al., 2008; Feng et al., 2019; Moiseeva et al., 2013; Wang et al., 2017; Xiong et al., 2017).

Additionally, gerotherapeutics are shown to regulate metabolism by targeting evolutionarily conserved nutrient‐sensing pathways across multiple species, primarily AMPK signaling by metformin, mTOR signaling by rapamycin and rapalogs, and insulin‐IGF1 signaling by acarbose (Barzilai et al., 2012; Tong et al., 2015). Glucose tolerance was improved with SGLT2i dapagliflozin while senolytics D + Q, acarbose reduced postprandial glucose levels and improved insulin sensitivity (Durak et al., 2018; Harrison et al., 2019; Sierra‐Ramirez et al., 2020). SGLT2i empagliflozin increased the AMP/ATP ratio in a cardiac injury model, induced renoprotection in diabetic mouse kidneys via inhibition of mTORC1 hyperactivation, while aspirin recapitulated metabolic effects of caloric restriction (Pietrocola et al., 2018; Tomita et al., 2020; H. Zhou et al., 2018).

### 3.2 Clinical results

#### 3.2.1 Mortality

To assess the effects of gerotherapeutics on mortality, we focused on all‐cause mortality and deaths from off‐target diseases, that is, those that were not the primary target for the drug (Table 3). SGLT2i demonstrated the most robust clinical evidence for reduction of death from any cause and CVD, in patients with or without T2D, heart failure, and chronic kidney disease (CKD) (Cavender et al., 2018; Heerspink et al., 2020; Neal et al., 2017; Packer et al., 2020; Perkovic et al., 2019). Clinical mortality data for metformin are less abundant, but UK Prospective Diabetes Study (UKPDS), an RCT that followed people with T2D for >10 years, found that metformin reduced all‐cause mortality compared to diet and other glucose‐lowering drugs (“Effect of Intensive Blood‐Glucose Control with Metformin on Complications in Overweight Patients with Type 2 Diabetes (UKPDS 34). UK Prospective Diabetes Study (UKPDS) Group,” 1998). Ample observational evidence supports survival benefits in people with diabetes treated with metformin, including when compared to people without diabetes (Bannister et al., 2014; Campbell et al., 2017; Jong et al., 2019; Ritsinger et al., 2020).

**TABLE 3:** Clinical evidence for candidate gerotherapeutics in targeting human healthspan and mortality in observational and interventional studies

Gerotherapeutics	Observational Healthspan	Interventional Healthspan	Observational Mortality	Interventional Mortality
SGLT−2 inhibitors	↓HF hospitalizations (HR = 0.61 [0.51–0.73]) (Kosiborod et al., 2017) ↓HF risk (HR = 0.72 [0.63–0.82]) (Cavender et al., 2018)	↓HF hospitalizations (HR = 0.70 [0.58–0.85]) (Packer et al., 2020) ↓CKD progression to ESRD (HR = 0.68 [0.54–0.86]) (Perkovic et al., 2019) ↓decline in GFR, ESRD, and death from renal causes (HR = 0.56 [0.45–0.68]) (Heerspink et al., 2020) ↓death from CV causes, nonfatal MI and CVA (HR = 0.86 [0.75–0.97]) (Neal et al., 2017)	↓all‐cause mortality (HR = 0.49 [0.41–0.57]) (Kosiborod et al., 2017)	↓all‐cause (HR = 0.68 [0.57–0.82]) and CV (HR = 0.62 [0.49–0.77]) mortality in pts with T2D (Zinman et al., 2015) ↓all‐cause mortality in patients with HF (HR = 0.83 [0.71–0.97]) (McMurray et al., 2019) ↓all‐cause mortality in patients with CKD (HR = 0.69 [0.53–0.88]) (Heerspink et al., 2020)
Metformin	Meta‐analysis: ↓CV mortality (OR 0.44 [0.34–0.57]) + CV events (OR 0.73 [0.59–0.90]) (K. Zhang et al., 2020) ↓ incident HTN (HR = 0.991 [0.989–0.994] per month of therapy) (C. Tseng, 2018) Meta‐analysis: ↓ abdominal aortic aneurysm progression (weighted mean difference: −0.83 [−1.38, −0.28] mm/year) (Yu et al., 2019) ↓ incident dementia (HR = 0.19 [0.04–0.85]) (Samaras et al., 2020) No Δ in cognition (Luchsinger et al., 2017) ↓ cognitive performance (OR = 2.23 [1.05, 4.75]) (Luchsinger et al., 2017) Meta‐analysis: ↓ overall cancer incidence (SRR = 0.69 [0.52–0.90]) (Gandini et al., 2014)	↓ T2D by 31% vs. plc in people with pre‐diabetes (Knowler et al., 2002) ↓ MI (RR = 0.61 [0.41–0.89]) and any macrovascular event (RR = 0.70 [0.52–0.95]) vs. conventional therapy (“Effect of Intensive Blood‐Glucose Control with Metformin on Complications in Overweight Patients with Type 2 Diabetes (UKPDS 34). UK Prospective Diabetes Study (UKPDS) Group,” 1998) ↓ CV events (HR = 0.54 [0.30–0.90]) vs. glipizide (Hong et al., 2013) Meta‐analysis: ↓cIMT (weighted mean difference = −0.049 [−0.095, −0.004] mm) (Y. Chen et al., 2020) ↑ memory in people with MCI in a pilot study (total recall in Selective Reminding Test 9.7 ± 8.5 vs. 5.3 ± 8.5, *p* = 0.02) (Luchsinger et al., 2016) Meta‐analysis: No Δ in cancer incidence (Stevens et al., 2012)	↑ survival in DM on metformin, vs. DM on SU (STR = 0.62 [0.58–0.66]) and non‐DM (STR = 0.85 [0.81–0.90]) (Bannister et al., 2014) ↓ risk for MACE (HR = 0.92 [0.85–0.997]) + all‐cause mortality (HR = 0.81 [0.71–0.92]), among people with history of MI (Ritsinger et al., 2020) ↓cancer mortality (HR 0.43 [0.23, 0.80]) (Landman et al., 2010)	↓ DM‐related mortality (RR = 0.58 [0.37–0.91]) and all‐cause mortality (RR = 0.64 [0.45–0.91]) vs. conventional therapy and superior to both SU +insulin in all‐cause mortality (*p* = 0.021) (“Effect of Intensive Blood‐Glucose Control with Metformin on Complications in Overweight Patients with Type 2 Diabetes (UKPDS 34). UK Prospective Diabetes Study (UKPDS) Group,” 1998) No Δ in all‐cause mortality (metformin vs. usual treatment: 1.1% [0.9–1.4%] vs. 1.3% [0.8–2.0%], *p* = 0.60); open‐label, 1‐year follow‐up (Cryer et al., 2005) Meta‐analysis: No Δ in all‐cause mortality (RR = 0.94 [0.79–1.12]) (Stevens et al., 2012)
Acarbose	↓ CV events (HR = 0.92 [0.85, 0.99]) vs. non‐acarbose (Ou et al., 2016) No Δ in CV outcomes (Ekström et al., 2016)	↓ T2D (RR = 0.82 (0.71–0.94)), no Δ in CV events in people with CAD +IGT (Holman et al., 2017) STOP‐NIDDM: ↓ T2D (HR = 0.75 [0.63–0.90]),↓ MACE ((HR = 0.51 [0.28–0.95]), ↓ MI (HR 0.09 [0.01–0.72]), ↓HTN (HR 0.66 [0.49–0.89]) (Chiasson et al., 2002, 2003)	No Δ in all‐cause mortality (Ekström et al., 2016)	No Δ in all‐cause mortality (Holman et al., 2017)
Rapamycin/Rapalogs	No applicable studies.	RAD001 vs. placebo min 20% ↑ in geom. mean titers of antibodies to min 2/3 influenza vaccine virus strains (Mannick et al., 2014) RAD001 + BEZ235 vs. plc ↓ self‐reported infections (*p* = 0.001) + ↑ type 1 IFN‐induced genes (Mannick et al., 2018) No Δ in cognition, grip strength or walking speed, but aimed to assess safety +feasibility (Kraig et al., 2018)	No applicable studies.	No applicable studies.
Methylene Blue	RCT analyzed as cohort study: people with mild AD, after 18 months ↓ progression of cognitive deterioration by ADAS‐cog (−3.14 [−5.32, −0.97] units), ADCS‐ADL (3.49 [0.66, 6.30] units) + ↓ MRI‐brain atrophy + ↑ brain glucose metabolism (Wilcock et al., 2018)	↓ progression of cognitive deterioration in moderate AD after 24 weeks (−5.42 [−9.44, −1.41] ADAS‐cog units) and mild and moderate AD after 50 weeks (−2.81 [−4.93, −0.70] and −5.18 [−9.72, −0.65] ADAS‐cog units, respectively) (Wischik et al., 2015)	No applicable studies	No applicable studies
ACEi/ARB	ACEi ↓ decline in BMD for black men (Rianon et al., 2017) Meta‐analysis: ↓ incident CRC (RR = 0.94 [0.89–0.98]) w/ ACEi or ARB (Dai et al., 2015)	↓DM with valsartan (HR = 0.86 [0.80–0.92]) (NAVIGATOR Study Group, 2010) ↓ nonfatal stroke (risk reduction = 27.8% [1.3–47.2%]), no Δ in MI +no Δ change in MMSE with candesartan vs. placebo (Lithell et al., 2003) Meta‐analysis: ACEi ↓stroke (RR = 0.80 [0.69–0.93]), CAD (RR = 0.87 [0.79–0.97]), HF (RR 0.79 [0.66–0.93]), ARB ↓stroke (RR 0.91 [0.86–0.97]), HF (RR = 0.90 [0.83–0.97]), no Δ in CAD (Thomopoulos et al., 2015) ↓ microalbuminuria (risk reduction = 12.5% [2–23%]) + ↓ decrease in GFR with enalapril vs placebo in people with DM (Ravid et al., 1998) ‐ Meta‐analysis: ACEi ↓ pneumonia vs. other BP meds/placebo (OR = 0.66 [0.55–0.80]) + vs. ARBs (OR = 0.69 [0.56–0.85]); both ACEi (OR = 0.73 [0.58 = 0.92]) + ARB (OR = 0.63 [0.40–1.00]) ↓ pneumonia‐related death (Caldeira et al., 2012) ACEi ↓decline in muscle strength + ↓ decline in gait speed in older women (Onder et al., 2002)	Meta‐analysis: No Δ in CRC mortality (Dai et al., 2015)	Post‐hoc analysis, Candesartan ↓ CV mortality vs. placebo (RR 0.71 [0.50–1.00], *p* = 0.049) + ↓ all‐cause mortality (RR = 0.73 [0.57–0.95]) (Lithell et al., 2004) No Δ in all‐cause mortality with candesartan vs. placebo (Lithell et al., 2003) Meta‐analysis: ACEi ↓ all‐cause mortality (HR = 0.90 [0.84–0.97] (Brugts et al., 2015)) Meta‐analysis: No Δ in all‐cause or CV mortality for either ACEi or ARB (Thomopoulos et al., 2015) Meta‐analysis: ↓ all‐cause mortality with ACEi (RR = 0.89 [0.80–1.00]; almost the entire difference explained by ↓ CV deaths), but not ARB (RR = 1.01 [0.96–1.06]), vs. placebo (Bangalore et al., 2016) Meta‐analysis: no Δ in all‐cause or CV mortality with ARB vs. placebo (Akioyamen et al., 2016)
Dasatinib + (quercetin)	No applicable studies	↑ 6‐min walk distance (+21 ± 28 m), 4‐m gait speed (+0.12 ± 0.2m/s), chair‐stands time (−2.2 ± 3s) in patients with IPF (Justice et al., 2019) ↓ senescent cell burden in adipose tissue and skin + ↓ circulating SASP in people with CKD (Hickson et al., 2019) In pts with ILD, by HRCT, 65% showed no progression in lung fibrosis and 39% showed no progression in total ILD, > historical controls; 23/31 participants analyzed (Martyanov et al., 2017)	No applicable studies	No applicable studies
Aspirin	No applicable studies.	No applicable studies.	No applicable studies	No applicable studies.
N‐Acetyl Cysteine	No applicable studies.	No applicable studies.	No applicable studies.	No applicable studies.

Except for ACEi/ARB, mortality data in appropriate dose/populations of the other evaluated drugs remain limited. One RCT of acarbose vs. placebo predefined the all‐cause and cardiovascular mortality as secondary outcomes and found no difference (Holman et al., 2017). Most studies analyzing mortality (and healthspan) with rapamycin/rapalogs focused on organ transplant recipients, a context that does not permit an assessment of gerotherapeutic benefits for several reasons: (1) the doses used were high and targeted to achieve immunosuppression, (2) these high‐risk patients are not representative of the general aging population, and (3) rapamycin/rapalogs were compared to other, more toxic immunosuppressants. Rapalogs are also used to coat intravascular stents, but systemic doses delivered by this route are merely a fraction of a single daily dose used to achieve immunomodulation (Wiemer et al., 2008). In small clinical trials, MB improved survival post cardiac surgery, owing to its vasoconstrictive effect, but it was not studied outside of the critical care setting (Levin et al., 2004). Numerous studies have investigated the role of aspirin in human lifespan and healthspan but, unlike animal studies, the administered doses were in the range at which aspirin displays anti‐platelet, not anti‐inflammatory activity. The use of anti‐inflammatory doses in humans would likely be prohibited by the risk of bleeding (ASCEND Study Collaborative Group, 2018; Hansson et al., 1998; McNeil et al., 2018; Ridker et al., 2005).

While abundant, clinical mortality data for ACEi/ARB in non‐high‐risk hypertensive populations (i.e., without heart failure, acute coronary syndromes, and organ transplants) are conflicted, at least in part due to marked variability in study populations, agents, and dosages used, comparators (placebo or active comparators), open‐label addition of other agents and achieved blood pressure goals (Hansson et al., 1999; Lithell et al., 2003, 2004; Oparil et al., 2013). Overall, the findings were predominantly negative, as illustrated in a meta‐analysis of 20 RCTs which found a borderline reduction in all‐cause mortality, driven by ACEi, while individual examination of the included studies shows that only 3 out of 20 showed mortality benefits, all with perindopril (van Vark et al., 2012).

#### 3.2.2 Human healthspan

In examining human healthspan, we focused on the ability of gerotherapeutics to prevent age‐related disease or reduce the progression of off‐target age‐related pathologies. SGLT2i, originally intended for glucose‐lowering, in RCTs demonstrated particularly strong and consistent cardioprotective and renoprotective effects, while metformin had beneficial effects across the widest range of healthspan outcomes (Heerspink et al., 2020; Neal et al., 2017; Perkovic et al., 2019; Wiviott et al., 2019). In RCTs, metformin prevented T2D and cardiovascular events (“Effect of Intensive Blood‐Glucose Control with Metformin on Complications in Overweight Patients with Type 2 Diabetes (UKPDS 34). UK Prospective Diabetes Study (UKPDS) Group,” 1998; Hong et al., 2013; Knowler et al., 2002). Pilot trials suggest that metformin may improve memory and executive function, while observational studies and meta‐analyses reported up to 81% reduction in incident dementia (Campbell et al., 2018; Cheng et al., 2014; Koenig et al., 2017; Luchsinger et al., 2017; Samaras et al., 2020; Zhou et al., 2020). By contrast, one small observational study reported impaired cognitive performance with metformin, but it did not adjust for glycemic control, duration of diabetes, or concomitant glucose‐lowering therapy (Moore et al., 2013). Another case–control study reported that metformin use was associated with increased odds for prevalent Alzheimer's disease but without consistent dose–response relationship or replication of findings with metformin monotherapy (Imfeld et al., 2012). Observational evidence further suggests that metformin may reduce overall cancer incidence by 31%, including colon, liver, pancreas, lung, and ovarian cancer (Gandini et al., 2014; Lee et al., 2011; Shi et al., 2019; Tseng, 2012; Xiao et al., 2020; Zhou et al., 2016).

Other drugs have been less studied in appropriate doses/populations, except for ACEi/ARB. In RCTs, acarbose prevented T2D in individuals with glucose intolerance (Chiasson et al., 2002; Holman et al., 2017). The STOP‐NIDDM RCT found a 49% reduction in major cardiovascular events with acarbose, while two RCTs in higher‐risk populations reported conflicted findings (Chiasson et al., 2003; Holman et al., 2017; Yun et al., 2016). Rapalogs have shown promise for boosting the immune response to vaccination and reducing respiratory infections in older individuals (Mannick et al., 2014, 2018). Recent phase 2b and phase 3 studies of RTB101 (also named BEZ235, dactolisib) reported a reduction in laboratory‐confirmed respiratory infections and upregulation of IFN‐induced antiviral genes in older participants. However, since RTB101 is not an FDA‐approved agent, we excluded these studies from grading (Mannick et al., 2021). MB and its derivatives show promise in reducing cognitive decline in mild and moderate Alzheimer's disease, while D+Q have shown beneficial effects in small open‐label pilot clinical trials in idiopathic pulmonary fibrosis and CKD (Hickson et al., 2019; Justice et al., 2019; Wilcock et al., 2018; Wischik et al., 2015).

ACEi/ARB prevented diabetes in RCTs (Bangalore et al., 2016; NAVIGATOR Study Group, 2010; Niklason et al., 2004; Os et al., 2008; Savarese et al., 2013; Song et al., 2012; Tocci et al., 2011). Most RCTs analyzing incident cardiovascular events in hypertensive populations were negative, but meta‐analyses reported cardioprotective effects (Akioyamen et al., 2016; Asselbergs et al., 2004; Brugts et al., 2015; DREAM Trial Investigators et al., 2008; NAVIGATOR Study Group, 2010; Haller et al., 2011; Lithell et al., 2003; Nakao et al., 2012; Thomopoulos et al., 2015). ACEi/ARB are also renoprotective, which is explained by the known beneficial effects of the renin–angiotensin–aldosterone system blockade on blood pressure and renal hemodynamics, rather than gerotherapeutic properties. Intriguingly, observational data suggest that centrally active ACEi may reduce cognitive decline, while non‐centrally active ACEi might increase the risk for dementia (Lithell et al., 2004; Sink et al., 2009). ACEi/ARB might also reduce the risk of pneumonia and pneumonia‐related death, reduce bone loss and hip fracture, and improve physical function (Caldeira et al., 2012; Onder et al., 2002; Rianon et al., 2017; Shea & Witham, 2020; Sumukadas et al., 2007).

## 4 DISCUSSION

The primary objective of the nascent field of geroscience is to translate the discoveries from basic research on the biology of aging to human studies and ultimately clinical care (Sierra et al., 2021). Current approaches to achieving this goal range from continuing robust research in preclinical models to testing candidate gerotherapeutics in clinical trials against individual age‐related diseases. Vigorous efforts into the development of possible phase‐III trials are needed and repurposing already FDA‐approved drugs represents a promising avenue offering accelerated evidence for geroscience at only a fraction of the cost and a faster rate of return than validating new compounds. A critical issue, therefore, is the identification of the best possible candidates for this purpose. Here, we identify several drugs that, like metformin, are relatively low‐cost molecular entities with a well‐established safety profile, representing possible candidates for inclusion in a geroscience‐guided clinical trial modeled after TAME, and aimed at probing whether a predefined cluster of age‐related diseases or well‐characterized loss of function can be significantly delayed using an approach based on targeting aging biology.

We provide a rigorous assessment of the literature concerning both preclinical and clinical status for several FDA‐approved potential gerotherapeutics. We developed a scoring scale that equally values (6 points each) the preclinical and clinical evidence supporting the claims for each of the nine potential gerotherapeutics selected. Surprisingly, with a score of 12/12, the best evidence was found for a relatively new class of drugs, the SGLT2i, which showed better promise than other known potential gerotherapeutics such as metformin, rapamycin, or acarbose.

The profound effects of SGLT2i on healthspan and lifespan in both animal models and humans may result from the pleiotropic effects beyond renal glucose and sodium handling, including improved mitochondrial function, restoration of autophagy, and promotion of ketogenesis‐dependent dampening of mTORC1 hyperactivation (Durak et al., 2018; Korbut et al., 2020; Tomita et al., 2020). We also identify several drugs that, like metformin, are relatively low‐cost molecular entities with a well‐established safety profile, representing possible candidates for inclusion in a geroscience‐guided clinical trial modeled after TAME, and aimed at probing whether a predefined cluster of age‐related diseases or well‐characterized loss of function can be significantly delayed using an approach based on targeting aging biology. Moreover, our geroscience‐guided literature‐driven approach is concordant with previously proposed data‐driven drug repurposing approaches using evidence from gene expression or model organism studies, with the overlap of rapamycin and senolytics identified as top candidate drugs across these (Dönertaş et al., 2018; Ziehm et al., 2017).

For our analysis, we decided to assign an equal number of points to knowledge derived from geroscience (i.e., preclinical) and to findings from humans (i.e., clinical—both observational and interventional). Although for translational purposes, knowledge from human studies is likely to have more weight than studies in rodents, we gave both equal values for several reasons: (1) The literature on potential gerotherapeutics in rodents is quite extensive and, while many of these studies are too recent to have been considered for clinical trials yet, their potential should not be dismissed based solely on their “novelty”; (2) some mouse studies, like the ITP, are quite robust, thus enhancing their predictive value as potential gerotherapeutics; (3) because of their highly controlled nature, studies in mice often have fewer caveats than similar studies in humans. Thus, while it could be argued that preclinical geroscience knowledge becomes less relevant if a drug has solid data on decreasing all‐cause mortality in humans, we decided it was not appropriate at this time to penalize potential drugs that have strong preclinical evidence.

In evaluating the preclinical data for specific drugs, we only considered rodent models. This approach excludes a sizable amount of literature on lifespan extension in lower organisms such as yeast, nematodes, and flies, but since the interventions have not yet been tested in mammals, approval for a trial in humans would likely take decades. Furthermore, in evaluating data on mouse longevity (a surrogate for all‐cause mortality) we gave more weight to studies that have been validated by the ITP, a well‐characterized, multicentered study that is widely accepted in the field to determine the translatability of gerotherapeutics, based on their rigor and their use of a genetically heterogeneous model that attempts to approximate the diversity in human populations. From the clinical side, we considered drugs that affect either all‐cause mortality (the most relevant parameter) or have a positive effect on unrelated diseases not intended to be the primary target of the drug. Thus, we were interested, for example, in a diabetes drug known to affect molecular pathways involved in aging, and that has been shown to also have a positive effect on incident off‐target age‐related diseases, such as cancer, dementia, or CVD.

Our approach restricted potential gerotherapeutics that affect hallmarks of aging without known effects on healthspan, as well as those that have not been tested for lifespan in animals. Two potential gerotherapeutics that failed in this test are colchicine (an anti‐inflammatory agent) and bisphosphonates (used in the treatment of osteoporosis). Interestingly, several studies show that bisphosphonates can decrease mortality even within critical care settings, where their action is clearly not related to their usual therapeutic use in osteoporosis. So, while these and possibly other FDA‐approved drugs might indeed show great potential as gerotherapeutics, they have not met our inclusion criteria, and their analysis is beyond the scope of this paper. The available data suggests that both colchicine and bisphosphonates should be rapidly tested in the ITP or in other centers, to confirm improved healthspan and lifespan in mammals.

In addition to the classes of drugs discussed above, another type of molecular entity that was not considered in our analysis deserves special mention; we did not consider nutraceuticals, several of which have indeed been shown to positively affect both healthspan and lifespan (e.g., NAD + replenishment). They not only lack robust pharmacological and clinical data, but also fail to serve our main purpose of identifying FDA‐approved drugs with the goal of repurposing them as gerotherapeutics that can reduce the rate of deterioration accompanying aging.

One important point that was also not considered in our analysis involves potential sexually dimorphic effects of gerotherapeutics. This is an important issue as we try to move geroscience into the clinical realm since many of the positive results published by the ITP are sex‐specific, increasing longevity either solely, or at least preferentially in one sex. For example, the ITP has clearly shown that acarbose has convincingly male‐specific effects on longevity (Nadon et al., 2017). Much more work is required in this domain, but we did not consider this variable because, in most human studies, data are adjusted for sex, so that potential differences are obscured, and in many cases, the size of the studies precludes the ability to perform a separate *bona fide* sex‐specific analysis. Another issue that is relevant for translating findings from geroscience‐guided animal studies into humans is the question of dosing. For instance, the doses of rapamycin or rapalogs used both in mice (ITP studies) and human (Mannick et al, 2014; Mannick et al, 2018; Mannick et al, 2021) studies are several times lower than doses commonly used in clinical practice to induce immunotolerance in transplant patients, or as an adjuvant to therapy in stage IV cancer. Furthermore, in human studies aimed at geroprotection (Mannick et al, 2014; Mannick et al, 2018; Mannick et al, 2021), these drugs have been used intermittently and/or for short periods, unlike in clinical practice where they are used chronically. As a result, especially in human studies, different dosage regimens result in different outcomes, *that is*, stimulation of the immune response in gerotherapeutic studies with intermittent exposure to low doses *vs*. immunosuppression in clinical settings with chronic administration of high doses. Investigating these dose‐specific differences will allow for minimizing side effects in gerotherapy clinical trials.

Recent advances in geroscience and aging biology research show that the hallmarks and pillars of aging described almost a decade ago interact strongly with each other, often in an additive or even a synergistic manner (Kennedy et al., 2014; Kulkarni et al., 2020; López‐Otín et al., 2013). It is therefore possible to imagine that future gerotherapies will encompass combinations of various gerotherapeutics (perhaps in lower doses and thus with better safety profile) or, in cases where a patient is already afflicted by a disease, a combination of a gerotherapeutic along with a disease‐specific intervention. For example, elimination of senescent cells in mice can improve the response to chemotherapy, both by further reducing tumor number and size, but also by decreasing side effects such as fatigue. Further research will be needed; however, since while some combinations (e.g., rapamycin +metformin) might result in further benefits than each drug alone, other combinations could result in either blunting of established geroprotective strategies (e.g., metformin or rapamycin with exercise) or worsening risk of toxicity often seen with polypharmacy in older adults (Strong et al., 2016; Walton et al., 2019).

Delaying aging to prevent multiple age‐related diseases is an obviously more exciting and promising approach than attacking one disease at a time, where because of comorbidities, the outcome is a no‐gain exchange of one disease for another. The benefits for health and fulfillment are obvious. However, there are also powerful political, economic, and societal gains to be attained, as framed in The Longevity Dividend (Olshansky, 2016). The economic value that can be derived from applying geroscience principles to healthcare has been recently reassessed showing that a slowdown in aging that increases life expectancy by just one year is worth $38 trillion a year, due to complementary gains in health and longevity (Scott et al., 2021). This is a very conservative estimate since the available data suggest that, for example, metformin has the potential to increase life span by ~3 years (Barzilai et al., 2016; Scott et al., 2021). Thus, targeting aging with gerotherapeutics offers potentially larger economic gains than eradicating individual diseases, and for both quality of life and economic reasons, we cannot afford to forfeit this opportunity for a truly transformational approach to healthcare.

## CONFLICT OF INTEREST

ASK is an employee of AbbVie, but his recent employment did not influence the work reviewed or presented in this manuscript. SA, DB, FS, GAK, and NB declare no competing interests.

## AUTHOR CONTRIBUTIONS

ASK, SA, and NB conceived the idea for the manuscript. All authors (ASK, SA, DB, FS, GAK, and NB) contributed to the literature search, writing, and editing of the manuscript.
